# Metformin in the Treatment of Amisulpride-Induced Hyperprolactinemia: A Clinical Trial

**DOI:** 10.3389/fnmol.2022.892477

**Published:** 2022-05-26

**Authors:** Cuifang Zhu, Ruofan Li, Mingliang Ju, Xudong Xiao, Ti-Fei Yuan, Zhixing Jin, Jing Zhao

**Affiliations:** Shanghai Mental Health Center, Shanghai Jiao Tong University School of Medicine, Shanghai, China

**Keywords:** schizophrenia, amisulpride, hyperprolactinemia, efficacy, metformin

## Abstract

**Objective:**

To evaluate the efficacy and safety of metformin in the treatment of amisulpride-induced hyperprolactinemia.

**Methods:**

A total of 86 schizophrenic patients who developed hyperprolactinemia after taking amisulpride were screened and randomly assigned to the metformin group (42 patients) and placebo group (44 patients) and followed up for eight weeks. The patients’ serum prolactin levels, blood glucose and lipids were measured at the baseline and the end of the intervention. The treatment emergent symptom scale (TESS) was also assessed.

**Results:**

After eight weeks of intervention, serum prolactin levels in the metformin group decreased from (1737.360 ± 626.918) mIU/L at baseline to (1618.625 ± 640.865) mIU/L, whereas serum prolactin levels in the placebo group increased from (2676.470 ± 1269.234) mIU/L at baseline to (2860.933 ± 1317.376) mIU/L. There was a significant difference in prolactin changes (F^covariance^ = 9.982, *P* = 0.002) between the two groups. There was no significant difference in the incidence of adverse drug reactions (*P* > 0.05) between the two groups.

**Conclusion:**

Metformin is able to improve amisulpride-induced hyperprolactinemia with its safety.

## Introduction

Prolactin (PRL) is a hormone mainly secreted by lactotroph cells of the anterior pituitary gland. PRL plays an important role in many biological effects, especially lactation during pregnancy (Capozzi et al., 2015). Hyperprolactinemia (HPRL) refers to an abnormally elevated level of serum PRL. HPRL causes a range of severe symptoms, including breast enlargement, galactorrhea, menstrual disorders, amenorrhea and infertility in female patients; breast feminization, decreased libido, erectile dysfunction and osteoporosis in male patients. Recent studies have shown that elevated PRL levels may also cause cognitive impairment and increase the risk of heart diseases (Montalvo et al., 2018; Toulis et al., 2018).

In pathological conditions, HPRL usually caused by prolactinomas, drugs and endocrinopathies such as primary hypothyroidism and primary adrenocortical insufficiency (Chahal and Schlechte, 2008; Capozzi et al., 2015). The most popular cause of non-tumor HPRL is drugs, especially antipsychotics. Nearly 70% of patients with schizophrenia develop antipsychotic-induced HRPL (Melmed et al., 2011). Dopamine released in the hypothalamus is important for regulating PRL as it can inhibit PRL synthesis and secretion. Antipsychotics with D2 antagonism properties can block dopamine receptors in the tuberoinfundibular pathway of the hypothalamus (Grigg et al., 2017). First-generation antipsychotics and some atypical antipsychotics (risperidone, paliperidone, and amisulpride) can easily lead to HPRL (Voicu et al., 2013). It was reported that the prevalence of HPRL was 59.6% in female schizophrenic patients and 40.0% in male schizophrenic patients (Wang et al., 2014).

Hyperprolactinemia (HPRL) has a significant impact on patients’ health and quality of life. Patients often discontinue their medications as a result, compromising the prognosis of the diseases. Therefore, coping with HPRL during treatment has become a challenge for clinicians. In several guidelines, asymptomatic antipsychotic-induced HRPL has no need to be treated, and clinicians should consider alternative medications for patients with symptomatic HPRL (Melmed et al., 2011; Halperin Rabinovich et al., 2013). Some studies suggested other medication treatments such as aripiprazole (Grigg et al., 2017). However, more research is needed to confirm these strategies.

Metformin is a potential treatment for antipsychotics-induced HRPL. In recent years, it is reported that (2.55–3 g/day) high-dose adjuvant metformin therapy can reduce antipsychotics-induced PRL elevation in patients with diabetes or prediabetes (Krysiak et al., 2016). However, previous studies did not have high level of evidence due to small sample sizes, the absence of a placebo group or patients with other diseases. Therefore, this study was proposed to investigate the efficacy and safety of metformin in the treatment of amisulpride-induced HPRL.

## Materials and Methods

### Study Design

The study used a double-blind, randomized, placebo-controlled design. The study was approved by the Ethics Committee of Shanghai Mental Health Center (ethical approval number: 2019-53). All patients signed an informed consent form to participate in the study.

The Metformin group was treated with metformin hydrochloride tablets (2 times/day, 250 mg/tablet, 2 tablets/dose, Shanghai Pharmaceuticals Sine) and the placebo group was treated with placebo (2 times/day, 2 tablets/dose, Shanghai Pharmaceuticals Sine). The doses used in this trial were based on previous studies (Wu et al., 2012; Zheng et al., 2017). There was no difference in appearance between placebo and metformin hydrochloride tablets. Both tablets were for oral administration for eight weeks. During the trial, the lifestyle (sleep schedule, diet and exercise) of all patients was the same. Subjects received regular medication during the study period. Their primary antipsychotic medications and doses were not changed. Depending on the subjects’ health status, combinations of other medications that did not affect serum PRL levels (e.g., antihypertensives, hypoglycemic agents, low-dose antidepressants, anxiolytics, or benzodiazepines, etc.) were allowed. The reason, dose, and start and stop times of the combined medications were recorded. Clinical and laboratory evaluation before and after the intervention included questionnaires on clinical impression (clinical global impression-severity of illness, CGI-SI) and side effects (treatment emergent symptom scale, TESS), and measurements of serum PRL, triglycerides, total cholesterol, high density lipoprotein (HDL), low density lipoprotein (LDL) and fasting blood glucose.

### Participants

Those who were hospitalized at Shanghai Mental Health Center from December 2019 to November 2021 and met the inclusion criteria were collected for the study.

Inclusion criteria: (1) meeting International Classification of Diseases 10 (ICD-10) diagnostic criteria for schizophrenia with positive and negative symptom scale scores > 60; (2) antipsychotic treatment with amisulpride alone; (3) age 18–60; (4) no gender limitation; (5) serum PRL > 1272 mIU/L.

Exclusion criteria: (1) meeting diagnostic criteria of other diseases in ICD-10; (2) organic diseases of the central nervous system; (3) serious physical diseases, especially endocrine diseases and gynecological diseases, pregnancy or lactation within two years; (4) taking other drugs affecting PRL levels; (5) severe impulsive, violent or suicidal behavior.

### Outcome Measurement

Serum PRL assay: Peripheral blood was collected using EDTA vacuum blood collection tubes and sent to the clinical laboratory of the hospital for testing on the same day (Equipment: Roche e601; method: electrochemiluminescence). Whole blood was centrifuged and the serum was separated.

Blood biochemical assay: Serum triglycerides, total cholesterol, high density lipoprotein (HDL), low density lipoprotein (LDL) and fasting blood glucose were measured at baseline and at the end of eight weeks of intervention. 5 mL of whole blood was drawn from the elbow vein at 7:00 a.m. on a fasting basis, and the serum was separated by centrifugation within 2 h after specimen collection. Blood glucose, total cholesterol, HDL and LDL were measured (Equipment: Beckman AU5811).

### Statistical Analysis

All analyses were conducted by using the Statistical Package for Social Sciences, version 23 (SPSS Inc, Chicago, Illinois). Descriptive analysis was performed of each efficacy index at each follow-up time point. The measurement data were shown as mean ± standard deviation (mean ± SD). Measurement data at different timepoints in the same group was compared by paired *t*-test. For measurement data with no significant difference between the two groups at the baseline, the *t*-test for independent samples was performed; for those with a significant difference at the baseline, the baseline data were used as covariates for analysis of covariance. The enumeration data were presented as percentages and chi-square tests were performed. Mann-Whitney U non-parametric tests were performed on data that do not conform to a normal distribution. *P* < 0.05 was considered statistically significant.

## Results

### Demographic Measurements

A total of 86 patients were included and randomly assigned to the metformin group and placebo group. Metformin group: 22 males and 20 females, 30–60 years old, 9–15 years of education, 7–45 years course of the disease, amisulpride dose 200–900 mg/day, duration of amisulpride 3.5–8 years; placebo group: 24 males and 20 females, 38–60 years old, 6–15 years of education, 14–40 years course of the disease, amisulpride dose 100–1000 mg, duration of amisulpride 3–8 years. There was no significant difference between the two groups in terms of age, education level, total disease duration, dose and duration of amisulpride, and gender composition ratio (*P* > 0.05) (Table 1).

**TABLE 1:** Demographic of 86 participants.

	Metformin	Placebo	t/x^2^	*P*
**Gender**				
*Male*	22	24	0.027	ns
*Female*	20	20		
Age (years)	53.33 ± 7.52	53.57 ± 6.03	–0.159	ns
Education (years)	13.00 ± 2.86	12.02 ± 1.77	1.893	ns
Course of disease (years)	29.67 ± 11.41	28.23 ± 5.30	0.745	ns
Amisulpride dose (mg/d)	490.48 ± 191.67	468.18 ± 187.73	0.545	ns
Duration of amisulpride (Years)	6.12 ± 0.97	5.74 ± 1.02	1.767	ns

*ns P > 0.05.*

### Changes in Prolactin

We next compared the PRL levels in the metformin group and placebo group. Because of the significant difference between the baselines of the two groups, here we used the analysis of covariance with PRL at the baselines as covariates. In all participants, the PRL of the placebo group was significantly higher than that of the metformin group after the intervention (F^a^ = 9.982, *P* = 0.002). It should be notable that the PRL of the metformin group showed a decreasing trend (from 1737.360 ± 626.918 to 1618.625 ± 640.865, *t* = 1.759, *P* = 0.086), while that of the placebo group showed an increasing trend (from 2676.470 ± 1269.234 to 2860.933 ± 1317.376, *t* = 1.866, *P* = 0.069). To figure out the effect of metformin if different genders, we compared the PRL levels in females and males separately. Similar to the above results, the PRL levels decreased in both females (F^a^ = 5.969, *P* = 0.02) and males (F^a^ = 12. 229, *P* < 0.001) (Table 2). These results indicate that metformin is able to inhibit the PRL increase in patients with antipsychotic-induced HPRL.

**TABLE 2:** Comparison of PRL levels between the two groups (mean ± SD) (mIU/L).

	Metformin	Placebo	t/F^a^	*P*
**Female**				
Participants	20	20		
Baseline	1947.367 ± 801.283	3705.489 ± 1195.010	–5.465	***
The end of 8 weeks	1798.310 ± 850.733	3923.382 ± 1222.330	5.969	*
**Male**				
Participants	22	24		
Baseline	1546.446 ± 325.950	1818.954 ± 379.508	–2.601	*
The end of 8 weeks	1455.276 ± 297.219	1975.560 ± 459.143	12.228	**
**Overall**				
Participants	42	44		
Baseline	1737.360 ± 626.918	2676.470 ± 1269.234	–4.380	***
The end of 8 weeks	1618.625 ± 640.865	2860.933 ± 1317.376	9.982	**

*^a^Analysis of covariance.*

*P < 0.05, **P < 0.01, ***P < 0.001.

### Blood Biochemical Index

Metformin is commonly used for the treatment of Type 2 Diabetes. Metformin is able to suppress the synthesis and storage of cholesterol to reduce blood lipids. By increasing glucose utilization, reducing insulin resistance and enhancing insulin sensitivity, metformin is helpful to reduce blood glucose. Metformin also has the potential side effect of hypoglycemia. Therefore, we measured the blood lipids and glucose before and after the intervention. After treatment, there were no statistically significant differences in indicators of blood lipids between the two groups (*P* > 0.05) (Table 3).

**TABLE 3:** Comparison of indexes of blood lipid between the two groups (mean ± SD, mmol/L).

	Metformin	Placebo	t	*P*
**Participants**	42	44		
**Triacylglycerol**				
Baseline	2.081 ± 0.892	2.009 ± 0.837	1.560	ns
The end of 8 weeks	1.817 ± 0.661	1.870 ± 0.933	0.725	ns
**Cholesterol**				
Baseline	4.553 ± 0.619	4.468 ± 0.892	0.510	ns
The end of 8 weeks	4.433 ± 0.338	4.353 ± 0.757	0.642	ns
**HDL**				
Baseline	1.249 ± 0.304	1.148 ± 0.246	1.685	ns
The end of 8 weeks	1.147 ± 0.285	1.172 ± 0.228	–0.456	ns
**LDL**				
Baseline	2.844 ± 0.337	2.874 ± 0.947	–0.196	ns
The end of 8 weeks	2.774 ± 0.230	2.699 ± 0.854	0.559	ns

*P < 0.05, **P < 0.01, ***P < 0.001, ns P > 0.05.

At the baseline, blood glucose in the two groups was in the abnormal distribution, and had significant differences in blood glucose between the two groups through Mann-Whitney U non-parametric test. Through the covariance analysis with baseline levels as covariances, the blood glucose showed a significant difference at the end of the intervention (Table 4). However, considering the normal fast blood glucose ranged from 3.9 to 6.1 mmol/L (China standard) (Jia et al., 2019), the changes in blood glucose of all patients were relatively minor and did not bring a significant risk of hypoglycemia.

**TABLE 4:** Comparison of blood glucose between the two groups [M (P25, P75), mmol/L].

	Metformin	Placebo		*P*
Participants	42	44		
Baseline	5.800 (5.123,7.400)	5.100(4.825,6.000)	*Z* = –2.459	**
The end of 8 weeks	5.935 (5.010,7.400)	5.050(4.600,6.000)	F^a^ = 4.172	*

*P < 0.05, **P < 0.01, ***P < 0.001, ns P > 0.05.

*^a^Analysis of covariance.*

### Psychotic Symptoms

The Clinical Global Impression Scale (CGI) is an overall rating scale for evaluating clinical impression of psychiatric diseases, which includes three parts: disease severity (SI), overall efficacy rating (GI) and efficacy index (EI). Here we used CGI-SI to investigate if metformin affects psychotics of the schizophrenia participants in the present trial. After eight weeks of treatment, there was no significant difference in CGI-SI score between the two groups, which indicated that the metformin did not affect the psychotics of the schizophrenia participants significantly (*P* > 0.05) (Table 5).

**TABLE 5:** Comparison of GCI-SI scores between the two groups (mean ± SD).

	Metformin	Placebo	t	*P*
Participants	42	44		
Baseline	3.500 ± 0.577	4.000 ± 0.695	–1.372	ns
The end of 8 weeks	3.500 ± 0.577	3.830 ± 0.592	–1.060	ns

*ns P > 0.05.*

### Adverse Drug Reactions

To investigate the safety of metformin in this trial, we compared the incidence rate of adverse drug reactions after taking metformin or placebo. Measured by the treatment emergent symptom scale (TESS) scale, there was no statistically significant difference of adverse reaction between the two groups (*P* > 0.05) (Table 6).

**TABLE 6:** Comparison of the incidence rate of adverse drug reactions between the two groups [Cases(%)].

	Metformin	Placebo	χ^2^	*P*
Participants	42	44		
Excitement or agitation	2(4.8)	1(2.3)	0.395	ns
Depression	0(0)	1(2.3)	0.96	ns
Insomnia	3(7.1)	4(9.1)	0.109	ns
Abnormal Liver Function	1(2.4)	2(4.5)	0.299	ns
Xerostomia	4(9.5)	3(6.8)	0.166	ns
Constipation	4(9.5)	3(6.8)	0.21	ns
Nausea and Vomiting	5(11.9)	4(9.1)	0.182	ns
Diarrhea	1(2.4)	2(4.5)	0.299	ns
Anorexia	3(7.1)	3(6.8)	0.003	ns

*P < 0.05, **P < 0.01, ***P < 0.001, ns P > 0.05.

## Discussion

In this trial, 86 schizophrenia patients were collected and assigned randomly to treatment with metformin or placebo. After eight weeks of intervention, we found that the PRL level of the metformin group decreased significantly compared to that of the placebo group. This treatment showed no significant adverse effects.

Prolactin (PRL) is mainly secreted by lactotroph cells of the anterior pituitary gland. Its synthesis and release are controlled by peptides, steroids and neurotransmitters. Dopamine released in the hypothalamus is important for regulating PRL as it can inhibit PRL synthesis and secretion. Antipsychotics with D2 antagonism properties can block dopamine receptors in the tuberoinfundibular pathway of the hypothalamus, and therefore induce HPRL (Besnard et al., 2014). Nearly 70% of patients with schizophrenia develop the antipsychotic-induced HPRL. The elevation degree of PRL depends on the affinity for D2 receptors of antipsychotic drugs. The incidence rate of HPRL is as high as 70%–100% in patients taking risperidone, 10%–40% in patients taking olanzapine and quetiapine, and less than 5% in those taking clozapine with no significant symptoms (Melkersson, 2005; Inder and Castle, 2011). It is found that both high and low doses of amisulpride can cause an increase in serum PRL (Andrade, 2013).

There is still no recognized effective treatment for antipsychotic-induced HPRL, which is a thorny problem in clinical treatment. The exploration of its treatment has been a hot spot of research in recent years, and the following strategies are mainly used: (1) Reduce the dosage of antipsychotic drugs. However, reducing the dosage of antipsychotic drugs may lead to the recurrence of the disease and is detrimental to the prognosis of patients. (2) Switching to other antipsychotic drugs with less possibility of causing HPRL (e.g., olanzapine, clozapine, quetiapine, etc.). However, switching to other drugs may not achieve the desired therapeutic effect and has the risk of causing other side effects such as sedation, weight gain, and even diabetes mellitus (Gianfrancesco et al., 2002; McQuade et al., 2004). (3) Combine with dopamine agonists, such as bromocriptine, amantadine, carte blanche, etc. However, there is evidence that dopamine agonists can exacerbate psychiatric symptoms or causes involuntary movements (Lertxundi et al., 2011). (4) Combine with some traditional Chinese medicine, such as Maiya, Sichuan Pepper, dandelions, Achyranthes Bidentata, Ziyin Rougan decoction, and Xiaoyao pills. However, the current studies lack high-level evidence because of their small samples (Yang et al., 2017). (5) Recent studies have shown that aripiprazole adjuvant therapy can reduce antipsychotic-induced HPRL with good tolerance (Raghuthaman et al., 2015; Jiang et al., 2018). It is also found that aripiprazole is effective in risperidone-induced HPRL, but it is not effective in HPRL induced by benzamide antipsychotics (amisulpride, sulpiride) (Chen et al., 2010). It is shown that the effectiveness of aripiprazole in reducing PRL increased with increasing dose, and the effectiveness was significantly higher in the patients with 10 and 20 mg/day than in patients with 5 mg/day (Chen et al., 2015). However, with increasing dose, its adverse effects increased, such as drowsiness and headache, which is harmful to patients’ compliance (Meng et al., 2015). On the other hand, Tomova and Caldwell (2016) suggested in their guidelines that aripiprazole agitates partially D2 receptors, leads to competitive receptor occupancy and may reduce the efficacy of the other antipsychotic drugs. Although the combination of more than two antipsychotics is common in clinical practice, based on systematic evaluation of safety and tolerability, the combination of antipsychotics should be the last option after the failure of monotherapy, conversion therapy, and combination therapy with non-antipsychotics (Gallego et al., 2012).

In recent years, it is reported that adjuvant metformin therapy is effective in antipsychotic-induced HPRL. Krysiak et al. found that metformin reduced plasma PRL levels in postmenopausal women with elevated PRL (Krysiak et al., 2021). In a previous study, 20 female patients with drug-induced HPRL received high-dose metformin treatment for six months (Krysiak et al., 2016). Their PRL levels and blood glucose reduced significantly, which indicated that metformin may benefit patients with drug-related HPRL. Several meta-analysis studies have evaluated the safety and efficacy of adjunct metformin therapy in the treatment of drug-induced HRPL. It was found that the PRL level decreased significantly with no significant increase in the incidence of side effects, which is consistent with our conclusion (Bo et al., 2016; Zheng et al., 2017).

In fact, metformin may have more benefits for patients with schizophrenia. For example, it is found that metformin is effective for antipsychotic-induced amenorrhea and weight gain in female patients (Wu et al., 2012). Furthermore, in patients with prolactinomas, metformin was reported to be able to reduce PRL levels and shrink the tumor. A hypothesis suggests that metformin may influence the activation of the LKB1/adenosine monophosphate-activated protein kinase (AMPK) pathway, which is involved in the synthesis and secretion of PRL (Liu et al., 2018).

The mechanisms of metformin in drug-induced HRPL are not figured out so far. There are some hypotheses based on current evidence. It is believed that metformin can inhibit the secretory function of overactive lactotrophs by targeting the pituitary, and therefore decrease the secretion of thyrotropin and PRL (Vigersky et al., 2006; Krysiak et al., 2015, 2021). This process is related to the dopaminergic regulation function of metformin (Vigersky et al., 2006; Cappelli et al., 2009). Dopamine is important for the synthesis and secretion of PRL. Drug-induced HRPL will lead to a decrease in the number or affinity of dopamine receptors. Metformin is able to improve the local dopamine action and therefore reduces the PRL level (Krysiak et al., 2016, 2018).

Long-term use of metformin may lead to some side effects, so we investigated the incidence of side effects in this study. We found no significant difference in the incidence of side effects in the metformin group compared with the placebo group. Meanwhile, there was no difference in blood lipids between the two groups. Although the blood glucose showed a significant difference between the two groups after the intervention, the blood glucose levels had relatively minor changes and had few risks of leading to hypoglycemia. These results indicated that the adjuvant metformin therapy has good security.

In conclusion, adjuvant metformin treatment can improve amisulpride-induced HPRL. This strategy has few adverse effects and can be promoted clinically. More research should be investigated in this field. For instance, In this research, participants were middle-aged and elder patients with a long duration of disease. It would be meaningful to investigate the efficacy and safety in different age groups in future studies. In addition, the effect of metformin on HPRL may not be fully revealed as the eight weeks intervention maybe not be long enough, and the decrease of PRL under the influence of drug intervention was significant but not satisfactory enough. In the future, the length of metformin intervention will be extended to six months to further evaluate the effect of long-term metformin administration on the PRL level.

## Data Availability Statement

The original contributions presented in the study are included in the article/supplementary material, further inquiries can be directed to the corresponding authors.

## Ethics Statement

The studies involving human participants were reviewed and approved by the Ethics Committee of Shanghai Mental Health Center (ethical approval number: 2019-53). The patients/participants provided their written informed consent to participate in this study.

## Author Contributions

CZ, JZ, and ZJ designed this study. CZ, RL, JZ, ZJ, MJ, and XX performed the experiments. CZ and RL analyzed the data and prepared the manuscript. T-FY revised it critically for important intellectual content and contributed to the final version of this manuscript. All authors have read and approved the manuscript.

## Conflict of Interest

The authors declare that the research was conducted in the absence of any commercial or financial relationships that could be construed as a potential conflict of interest.

## Publisher’s Note

All claims expressed in this article are solely those of the authors and do not necessarily represent those of their affiliated organizations, or those of the publisher, the editors and the reviewers. Any product that may be evaluated in this article, or claim that may be made by its manufacturer, is not guaranteed or endorsed by the publisher.
